# Prevalence of common mental health disorders in military veterans: using primary healthcare data

**DOI:** 10.1136/bmjmilitary-2021-002045

**Published:** 2022-01-18

**Authors:** Alan Finnegan, R Randles

**Affiliations:** Westminster Centre for Research in Veterans, University of Chester Faculty of Health and Social Care, Chester, UK

**Keywords:** mental health, public health, primary care

## Abstract

**Introduction:**

Serving military personnel and military veterans have been identified as having a high prevalence of mental disorders. Since 1985, UK patients’ primary healthcare (PHC) medical records contain Read Codes (now being replaced by Systematized Nomenclature of Medicine - Clinical Terms (SNOMED CT) codes) that mark characteristics such as diagnosis, ethnicity and therapeutic interventions. This English study accesses a cohort profile of British Armed Forces veterans to examine the diagnosed common mental disorders by using PHC records.

**Methods:**

This analysis has been drawn from initiatives with PHC practices in the Northwest of England to increase veteran registration in general practice. Demographic data were collected including gender, age and marital status. Data were also collected on common mental health disorders associated with the Armed Forces.

**Result:**

2449 veteran PHC records were analysed. 38% (N=938) of veterans in this cohort had a code on their medical record for common mental health disorders. The highest disorder prevalence was depression (17.8%, N=437), followed by alcohol misuse (17.3%, N=423) and anxiety (15.0%, N=367). Lower disorder prevalence was seen across post-traumatic stress disorder (PTSD) (3.4%, N=83), dementia (1.8%, N=45) and substance misuse (0.8%, N=19). Female veterans had a higher prevalence of mental disorders than their male counterparts, while men a higher prevalence of PTSD; however, the gender difference in the latter was not significant (p>0.05).

**Conclusion:**

The SNOMED searches do not detail why certain groups had higher recordings of certain disorders. A future study that accesses the PHC written medical notes would prove enlightening to specifically identify what situational factors are having the most impact on the veteran population. The results from a sizeable English veteran population provide information that should be considered in developing veteran-specific clinical provision, educational syllabus and policy.

**Key messages**

- Thirty-eight per cent of veterans had a code on their medical record for a common mental health disorder.
- The highest disorder prevalence was depression at 18%, followed by alcohol misuse at 17%, anxiety 15%, post-traumatic stress disorder was 3% and dementia 2%.
- The SNOMED searches do not detail why certain groups had higher recordings of certain disorders.
- Female veterans have a higher prevalence of common mental disorders than their male counterparts.
- The National Health Service England and Royal College of General Practitioners need to identify if there is a mechanism for increasing primary healthcare veteran registration to provide a more reliable source of data.

## Introduction

Serving military personnel have been identified as having a high prevalence of mental disorders with the likelihood of common mental disorders (CMDs) being approximately doubled.^1^ In the British Armed Forces, depression is the most common mental health (MH) disorder, with the Ministry of Defence (MoD) reporting that 12.7% of the British Armed Forces were seen in military healthcare for an MH-related reason with 2.7% being seen by a specialist MH clinician.^2^ There is estimated to be 2.4 million UK Armed Forces veterans, which make up an approximated 5% of the total population.^3^ Within this veteran population, 89% are male, 60% are aged 65 years and over, and 62% are married.^3^ Military veterans have a higher prevalence of CMD including anxiety, depression,^4 5^ post-traumatic stress disorder (PTSD) and alcohol misuse than the general population,^6^ and these rates are higher for those with combat experience.^7^

To improve MH support for veterans, National Health Service England (NHSE) launched Op COURAGE: the Veterans Mental Health and Wellbeing Service, which aims to facilitate priority access to veteran-specific services for service-related problems.^8^ The effectiveness of this initiative in part requires veterans to be correctly identified and registered with a primary healthcare (PHC) practice. Since 1985, UK patients’ PHC medical records contain Read Codes (now being replaced by Systematized Nomenclature of Medicine - Clinical Terms SNOMED CT codes) that mark characteristics such as diagnosis, ethnicity and therapeutic interventions, and a code to annotate ‘military veteran’.^9^ One means to therefore estimate the level of MH disorders in the veteran population is through the PHC medical records. However, veterans typically have poor help-seeking behaviour for mental disorders; barriers include stigma, military culture of stoicism and concerns surrounding understanding by healthcare staff.^10^ It may take reaching crisis point to seek help, leading to higher rates of PTSD.^11^ Research indicates that only 8% of the veteran population are correctly registered; resulting in a potential lack of support for their specialised MH needs.^12^ The Royal College of General Practitioners (RCGP) launched a ‘veteran-friendly practice’ accreditation in England to improve the recording and support of veterans within PHC; by October 2021, there were 1050 (from approximately 7000) practices accredited,^13^ yet the number of correctly registered veterans remains low. This paper reports the levels of military veterans with CMDs using data drawn from PHC medical records. The authors are unaware of any similar English study with a cohort profile of English veterans.

## Aim and objectives

The aim was to identify the prevalence of CMD within the English veteran population. The objectives are to: (a) identify trends regarding veterans’ age, gender and marital status; (b) identify the prevalence of dementia, PTSD, depression, anxiety, alcohol misuse and substance misuse.

## Methodology

PHC staff provided the data from PHC patient medical records. Read/SNOMED codes were used. These codes provide a consistent vocabulary for PHC staff to record factors such as characteristics, diagnosis and pharmacological treatments onto a patient’s medical record. For example, if a patient is diagnosed with depression, a code will appear on their record for ‘depression’. There are many different codes available, and it is to the discretion of the staff member who is recording the information to choose the appropriate code. The dependent variable was the SNOMED (code 753651000000107) or Read Code (code 13Ji) for ‘military veteran’ which was then correlated with the common military MH^8^ recording of the codes for depression, anxiety, PTSD, alcohol misuse, substance abuse and the physical disorder of dementia. Practices were asked to include codes related specifically to these disorders. There was direction to try and ensure consistency as there were numerous codes that were used. For example, ‘depression’ included depressive episodes and depressive disorder, and anxiety often being coded along with depression as ‘anxiety with depression’ or ‘mixed anxiety and depression’ as well as its own code of ‘anxiety disorder’. PTSD was only coded as ‘post-traumatic stress disorder’. Substance abuse was often coded more specifically such as ‘heroin addiction’ or ‘opioid dependence’, all substance dependencies and addictions were asked to be included within the search. In addition, for alcohol this was coded as ‘alcohol abuse’ or ‘chronic alcoholism’ or simply ‘alcohol’. Dementia was coded as ‘dementia in Alzheimer’s’ or ‘Alzheimer’s disease’ or simply ‘dementia’. Significant MH disorders such as bipolar and schizophrenia should have restricted people with these problems from enlisting and were therefore excluded. Data were received in an anonymised and amalgamated form. Being aware of the considerable pressures on PHC, the search strategy was designed to maximise the data that could be collected while being cognisant of other demands and priorities on PHC staff. Therefore, the searches were restricted to common military MH disorders and basic demographical data (age, gender and marital status). The PHCs were financially remunerated for their time. Data were inputted into an SPSS database (Version 27) for analysis where descriptive statistics were identified, followed by inferential analyses of the relationships between MH disorders and demographical data.

## Method

The authors worked with 16 PHCs across Cheshire and Lancashire with a total patient population of 178,568 to improve their veteran patient registration. Drawing on an estimated veteran population of 5% per PHC practice in Northwest England meant that at the commencement of these projects, 9.1% (N=781) of veterans were correctly coded; increasing to 28.7% (N=2449) by the time the project ended. This final figure provided the data for this paper, and the information was collected in March 2021. The research was funded by NHSE and Forces in Mind Trust (FiMT).

## Results

**Figure 1:**
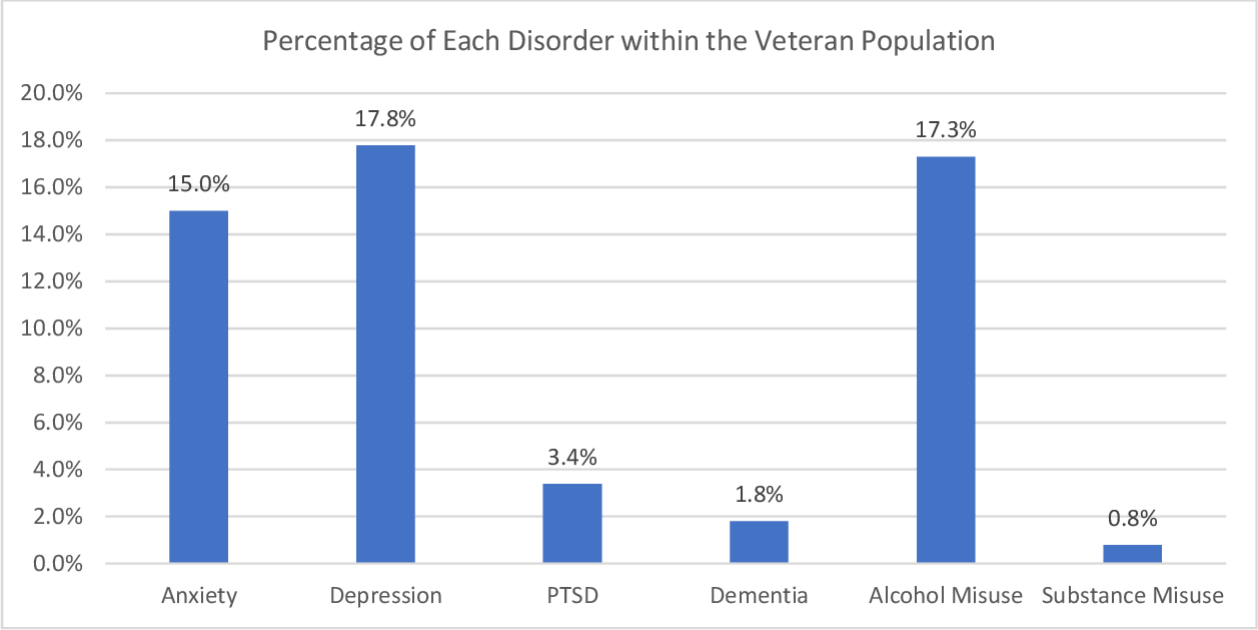
Percentage of each disorder present within the veteran population. PTSD, post-traumatic stress disorder.

From 2449 veteran PHC records, 88% (N=2147) were male and 12% (N=302) female. The average age was 62 years with a SD of 18. The youngest veteran was 16 years old and the oldest 99 years. The mode of the veterans was the age group 58–67 years, with 20% (N=490) belonging to this age group. For 68.4% (N=1675) of veterans, marital status was unknown. Of the remaining 774 veterans, 67% (N=518) were married, 20% (N=154) single, 5% (N=38) widowed, 4% (N=33) divorced, 2% (N=19) cohabiting, 1% (N=7) separated and 1% (N=5) were in a common law partnership. Thirty-eight per cent (N=938) of the veterans had a code on their medical record for PTSD, depression, anxiety, alcohol abuse, substance misuse or dementia. Furthermore, there was some comorbidity with veterans with more than one disorder. The highest disorder prevalence was depression (17.8%, N=437), followed by alcohol misuse (17.3%, N=423) and anxiety (15.0%, N=367); while PTSD was (3.4%, N=83), dementia (1.8%, N=45) and substance misuse (0.8%, N=19) (see Figure 1).

**Figure 2:**
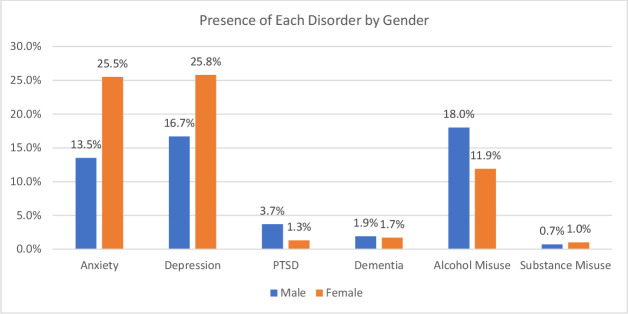
Percentage of each disorder present split by gender. PTSD, post-traumatic stress disorder.

A total of 37.5% men (N=805) and 44.0% women (N=133) had a code on their record for a mental disorder. The relationship between gender and presence of a mental disorder was found to be significant (X^2^ (1, N=2449)=4.5, p<0.05) suggesting that female veterans have a higher prevalence of mental disorders than their male counterparts as is consistent with the general population.^14^ Figure 2 shows the prevalence of each disorder by gender. Women had a higher prevalence than men for both anxiety (25.5%, N=77) and depression (25.8%, N=78), which may be due to poorer help-seeking behaviour from the male veteran population.^10^ The relationship between anxiety and gender was also found to be significant (X^2^ (1, N=2449)=29, p<0.01), as was the relationship between depression and gender (X^2^ (1, N=2449)=14.4, p<0.01). Women also had a higher prevalence of substance misuse (1.0%, N=3), however this difference was not significant (X^2^ (1, N=2449)=0.01, p>0.05). In contrast, men had a higher prevalence of PTSD (3.7%, N=79), however the difference was not significant (X^2^ (1, N=2449)=3.8, p>0.05). Men also had a higher prevalence of dementia (1.9%, N=40), but again the difference was not significant (X^2^ (1, N=2449)=0.001, p>0.05) (see Figure 2).

**Figure 3:**
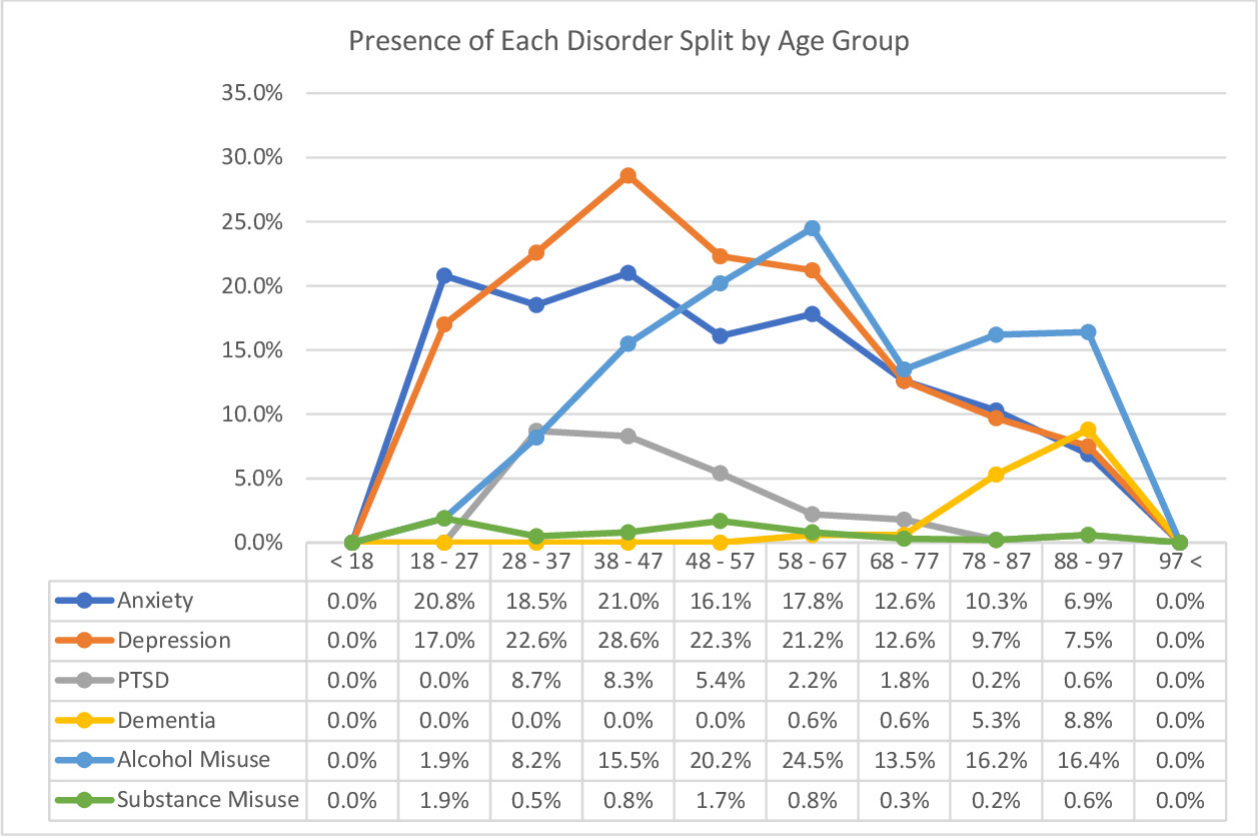
Percentage of each disorder present split by age group. PTSD, post-traumatic stress disorder.

The individual age with the highest percentage of mental disorders was 41 years, meaning 66.7% (N=10) of those aged 41 years had a mental disorder coded on their record. However, the age group with the highest number of veterans with mental disorders, and therefore the mode, was 48 years (N=29). Of those with mental disorders, the average age was 61 years, and the median age 60 years. The age group with the highest percentage of mental disorders was the group 38–47 years (44.4%, N=112), followed closely by the group 58–67 years (43.9%, N=215) and the group 48–57 years (43.8%, N=212). This percentage then decreased for the group 68–77 years (30.5%, N=99), climbing gradually thereafter, likely due to the presence of dementia. The difference between the age groups on having a mental disorder present was found to be significant (X^2^ (9, N=2449)=40, p<0.01) (see Figure 3).

The results indicated the presence of each disorder within the different age groups. Anxiety was the most prevalent among the age group 38–47 years (21.0%, N=53) closely followed by the 18–27 years (20.8%, N=11) suggesting anxiety has a higher prevalence among younger age groups in the veteran population. The difference between these groups in regard to anxiety prevalence was found to be significant (X^2^ (9, N=2449)=32.7, p<0.01). Depression was also highest among the age group 38–47 years (28.6%, N=72), likely due to the high comorbidity of these disorders; but the second highest prevalence was found in the group 28–37 years (22.6%, N=44). These differences were also found to be significant (X^2^ (9, N=2449)=73.9, p<0.01).

For PTSD, the highest prevalence was among the age group 28–37 years (8.7%, N=17), closely followed by the age group 38–47 years (8.3%, N=21), with significant differences between the groups (X^2^ (9, N=2449)=66.6, p<0.01). Dementia, as expected, had a higher prevalence among the older age groups with prevalence highest in the group 88–97 years (8.8%, N=14)—the differences again being significant (X^2^ (9, N=2449)=101.2, p<0.01). Alcohol misuse had the highest prevalence among the age group 58–67 years (24.5%, N=120), followed by the age group 48–57 years (20.2%, N=98), these differences were also significant (X^2^ (9, N=2449)=45.9, p<0.01). Finally, for substance misuse, the highest prevalence was among the age group 18–27 years (1.9%, N=1), closely followed by the age group 48–57 years (1.7%, N=8). However, the differences between these groups, for substance misuse, were not significant (X^2^ (9, N=2449)=8.9, p>0.05) (see Figure 3).

## Discussion

Using PHC medical records to identify the prevalence of common MH disorders within the veteran population is severely hindered by the low percentage of veterans with the correct Read/SNOMED code applied (currently estimated to be under 10%^12 15^). The authors were part of NHSE and FiMT-funded programmes to improve veteran registration and PHC staff awareness; consequently, this study was based on an average of 26.7% per practice (range 5.0%–54.1%). Searches were limited to the known common military MH disorders of PTSD, depression, anxiety, alcohol misuse, substance abuse and the physical disorder of dementia. If all MH disorders had been included, the overall total would have been higher.

Within this study, demographics from a gender perspective aligned to this known non-parametric population with 88% being male. Forty-six per cent of the veterans were aged 65 years and over, significantly lower than the MoD estimate of 60% for this age group.^3^ The data from this study indicated that approximately 70% of veterans were in a relationship (67% married) and only 4% divorced. These are significantly lower than the general population and may be due to factors such as medical records not being updated. Age does indicate that, in particular, the more elderly veterans are not correctly registered with their general practitioner (GP) practice. There are multiple reasons for this, such as veterans being unaware of the 1-day inclusion criteria and therefore being unaware of their veteran status and never declaring as such. This could extend to those more vulnerable members of the population in care and residential homes, or with dementia, who may be reliant on others for much of their communication with PHC.

The overall rate of MH in the UK Armed Forces was found to be broadly comparable with the UK general population.^16^ For the veteran cohort within this research study, depression, anxiety and alcohol misuse were the leading MH disorders. However, the levels of depression were lower than identified within the Defence Medical Services and the national population. Within this study, level of depression was 17.8%, while in the general population this stands at 21%, and for serving personnel at 32%; though prior to the isolation of COVID-19, the level in the general population was lower at 19%.^2 17^ Both within the MoD and the general population, mild to moderate depression is predominately due to situational stressors such as relationship problems, employment and financial problems.^18 19^ The more elderly group may be retired, and for those veterans who served a full career, the military pension is highly competitive; this may in part explain why levels of anxiety and depression reduced notably as the veteran got older. Depression and anxiety are also often linked (comorbidity) and some research studies may place an emphasis on the depressed element. There is also the issue that GPs will often not identify depression or record depression on a patient’s medical record due to the direction of their patients who are concerned about the impact of a MH diagnosis on their future career options, or other everyday issues such as higher travel insurance costs.^20^

Depression and anxiety were both significantly higher in women, which warrants further investigation. Previous studies have indicated that GPs were more likely to see female patients on a regular basis, which may help indicate why such issues may be picked up more often in women.^21^ Furthermore, the Armed Forces may be seen as having a more ‘macho’ culture than some other careers, which may lead to poor help-seeking behaviour and concerns of being labelled ‘weak’—potentially another factor in a lower level of depression and anxiety being picked up in male veterans.^10^ However, there is evidence that help-seeking behaviour is increasing among both serving and ex-serving personnel.^22^ In terms of age difference, the study revealed that anxiety in those aged 18–27 years old accounted for 21% (N=11) of cases. By their very essence, this age group will include young early service leavers. As the peak of the last major deployments (and therefore associated operationally related MH problems) was over 10 years ago, their anxiety may be due to their current living situations and potentially not related to their military service. However, anxiety and depression were highest in the age group 38–47 years, a possible reason being that veterans served longer and had greater difficulties in transitioning from the Armed Forces in relation to accommodation, finance, housing and employment. There may also have been adjustment issues for their spouse and children including schooling. But the coding of the veterans’ medical records does not capture this information.

PTSD in a combined sample of veterans and serving personnel was found to be 4% in 2004/2006 and 2007/2009 but had risen to 6% in 2014/2016.^23^ This compares with a rate of 4.4% within the civilian population.^23^ The prevalence of PTSD is not consistent across these groups, however, with serving personnel having a prevalence of 4.8% and veterans significantly higher at 7.4%.^23^ The PTSD levels in this study were comparable at 3.4% (N=83). The results, however, may be inflated as PTSD may remain on a patient record even when the patient is deemed to have received successful treatment, and unless the date of the last positive assessment or treatment is recorded then diagnosis will remain in the patient’s medical record. Ideally, an inclusion criterion such as a positive assessment and affirmation of the condition in the last 5 years would help rectify this concern. Many studies show male veterans have significantly higher levels of PTSD but that was not the case in this study. In the UK, frontline troops are identified as having 17% compared with 4% on non-frontline troops which can be attributed to their role in the military and the increased likelihood that they have been in contact (combat) situations.^23^ However, the average age group of those recorded in GP practices in this study at 38–47 years is significantly different to the attributed average age of veterans. Furthermore, the highest prevalence was 28–37 years old, which would include those who had deployed to Afghanistan (2004–2014) and Iraq (2001–2012).

The levels of listed alcohol misuse are of concern but significantly less than that often associated with the military. A potential reason is that veterans may be consuming more than the recommend weekly average; but they are functioning very well, have no concerns about their behaviour and are therefore not raising it as an issue with their GP. Even when they have, as it is not causing any problems, then the GP has not recorded the detail. There are differences between genders with men more likely to be diagnosed with an alcohol-related problem. Potentially harmful alcohol misuse remains a common behavioural problem but has declined steadily from 16% in 2004/2006 to 10% in 2014/2016.^23^ Within these studies on veteran population, the highest was in the age group 58–67 years. A combination of factors such as the progressive nature of alcohol misuse, potentially being retired and that the records may be related to a diagnosis some years before are all potential factors.

Substance abuse was higher in women but listed in only 1% of cases and not statistically significant. The military’s regular random drug tests are a deterrent and this may have transitioned into veteran behaviour. It could also be due to under-reporting and that this is a more elderly population. Substance misuse was most prevalent in the group 18–27 years old, so again the early service leavers. There may also be a sense of shame from those who were discharged due to substance abuse and therefore they may not want to declare their veteran status.

Dementia was notable in the 78 years old and above cluster and most prevalent in the age group 88–97 years. As 60% of the veteran population are over 65 years and 50% over 70 years old, the authors had expected to see higher levels of dementia. As the leading cause of death within the UK, with a population estimate of 7.1% then the 1.8% within this study appears low.^24^ Recent studies have indicated that dementia rates in the veteran population are not higher than the general population.^25^ The low rate in this study is likely to be under-recording due to PHC being unaware of the veteran status and therefore does not have the correct military veteran code attached to their records. The NHSE and RCGP would need to identify if there is a mechanism for increasing registration for this group which would provide a more reliable source of data. It would be reasonable to anticipate that large numbers of veterans are residing in care/residential homes and a means of connecting with them to get them registered is required. In addition to ensuring this can be used to provide the best clinical care, then it also has important ramifications for non-statutory and third-sector organisations that have considerable resources to assist all of the veteran population. There is also the issue of UK women living on average to an older age than men (83 compared with 79 years), and veterans’ spouses being left socially isolated, with financial difficulties and increasing dementia issues without their needs to be correctly identified, assessed and supported.

### Limitations

Although Read/SNOMED codes were created to ensure a consistent vocabulary is used across medical records, there are issues with consistent coding of information. Within this study, the authors chose to use codes and subtypes of the disorders, in an attempt to show a more inclusive picture. However, there is likely still coding issues where some subtypes could classify as ‘depression’ or ‘anxiety’ but have not been included. For example, phobias were not included in this project nor was obsessive-compulsive disorder, both of which could be seen as a subtype of anxiety disorder. Further, disorders such as ‘seasonal affective disorder’ were not included, which could be seen as a depressive subtype. Furthermore, within some practices, when a patient presented with anxiety and depression, this was grouped as one code of ‘mixed anxiety and depression’ or ‘anxiety with depression’ showing some inconsistencies with coding as both of these different codes were often used within the same practices. Therefore, there are numerous consistency problems with the Read/SNOMED codes which may have affected the results of this project. The results do not inform as to the causes of the MH disorders and are, therefore, unclear as to whether the disorders were service related. In addition, to be classified as a ‘veteran’, an individual only has to serve for 1 day, this includes training, national service and reservists.^26^ Essentially, this could mean that there may be individuals who had left after a significantly short period and therefore are likely to be unaffected by the service and the MH disorder not attributable to their time in the military. Furthermore, the coding of an MH disorder can indicate a lifetime prevalence. The conditions have been added onto the patient’s medical record, but without interrogation of the notes or asking the doctor or patient, it can be unclear if the condition has been resolved or is ongoing. Therefore, the results of the study could be potentially inflated. The authors are aware of previously raised concerns with MH disorder such as schizophrenia, where a diagnosis in the patient’s teens can remain with that person for their entire life, irrespective of any future manifestation or symptoms. That one patient was listed as a veteran and was 16 years old indicate that there was the potential for some incorrect coding.

## Conclusion

The searches were limited to the MH disorders commonly associated with the Armed Forces and veterans with PTSD, depression, anxiety, alcohol misuse, substance abuse and the physical disorder of dementia. This was a deliberate strategy with a key determinant being not to overwhelm the PHC staff completing the searches. This requirement was discussed with each of the 16 participating practices before the programmes started and it was clear that the association between the rationale for these searches was embraced by the staff and therefore helped compliance with conducting the data collection.

The SNOMED searches do not detail why certain groups had higher recordings of a certain disorder and a future study that accesses the written medical notes would prove enlightening to specifically identify what situational factors were having the most impact on the veteran population. As far as the authors can determine, this is the first published article outlining the English veteran morbidity using PHC medical records. As such, the results from a sizeable English population provide information that should be considered in developing veteran-specific clinical provision, educational syllabus and policy.

## Data Availability

Data are available upon reasonable request. Should anyone wish to have access to the data, they can request as such.
